# Beam Scanning with Ultra‐Low Sidelobes and In‐Band Ultra‐Low Scattering Characteristics Empowered by Single Space‐Time‐Coding Radiation‐Scattering Metasurface

**DOI:** 10.1002/advs.202413429

**Published:** 2025-01-29

**Authors:** Lixin Jiang, Yongfeng Li, Hao Yang, Mingbao Yan, Jinming Jiang, Yunwei Zhang, Zhe Qin, Wanwan Yang, Hongya Chen, Yongqiang Pang, Zhihao Guo, Lin Zheng, Jiafu Wang, Shaobo Qu

**Affiliations:** ^1^ Shaanxi Key Laboratory of Artificially Structured Functional Materials and Devices Airforce Engineering University Xi'an Shaanxi 710051 China; ^2^ Electronic Materials Research Laboratory Key Laboratory of Ministry of Education Xi'an Jiao‐tong University Xi'an Shaanxi 710049 China

**Keywords:** radiation‐scattering, space‐time‐coding metasurface

## Abstract

The integrated modulation of radiation and scattering provides an unprecedented opportunity to reduce the number of electromagnetic (EM) apertures in the platform while simultaneously enhancing communication and stealth performance. Nevertheless, achieving full‐polarization, arbitrary amplitude, and phase modulation of radiation scattering remains a challenge. In this paper, a strategy that realizes space‐time coding of radiation scattering within the same frequency band, which enables the simultaneous and independent modulation of amplitude and phase, is proposed. To address the limitations of the high sideband levels (SBLs) of conventional space‐time‐coding metasurfaces, a strategy comprising nonuniform modulation periods and stochastic coding is proposed. Consequently, beam scanning with ultra‐low sidelobe levels (SLLs) and suppressed SBLs is achieved in the radiation mode (RM). In scattering mode (SM), in‐band low scattering characteristics are achieved within the same operating frequency band as RM. A prototype of a space‐time‐coding radiation‐scattering metasurface (STCRSM) is fabricated and the aforementioned functionalities are validated by measurements. Furthermore, the proposed strategy does not necessitate the utilization of optimization algorithms and exhibits low SLLs and low SBLs, which will make it flourish in RF stealth applications, such as covert communication systems.

## Graphical abstract

A novel strategy for integrating radiation and scattering is proposed. The simultaneous and independent modulation of amplitude and phase is achieved by employing space‐time coding of joint amplitude and phase of radiation scattering. Hence, beam scanning with ultra‐low sidelobe and in‐band ultra‐low scattering characteristics are realized. Furthermore, the proposed strategy of stochastic coding and nonuniform modulation enables ultra‐low sideband levels.

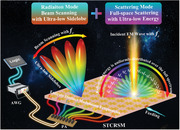

## 1 Introduction

The integration of electronic information systems places greater demands on the modulation of radiation and scattering characteristics. It opens up the possibility of simultaneous signal transmission and signal relaying. Furthermore, this integrated design, which consolidates numerous discrete electromagnetic (EM) functional units, can significantly reduce the number of EM devices on the platform while simultaneously enhancing the platform's communication and stealth performance. A number of studies have been devoted to the simultaneous manipulation of radiation and scattering characteristics, including the optimization of the antenna structure,^[^ ^1^ ^]^ or loading the antenna structure with impedance structures,^[^ ^2^ ^]^ frequency selective surfaces,^[^ ^3^ ^]^ and absorbing materials.^[^ ^4^, ^5^ ^]^ However, these efforts tend to be static and single‐polarized, which frequently encounter mutual constraints on radiation and scattering performance,^[^ ^6^ ^]^ as well as increased complexity of the system. In order to achieve highly integrated and adaptive information systems, metasurfaces have been incorporated into antenna design, which will offer unparalleled potential for the modulation of radiation and scattering.

Metasurfaces^[^ ^7^, ^8^, ^9^ ^]^ are two‐dimensional arrays of subwavelength meta‐atoms that enable a range of modulations on the scattering characteristics of EM waves, including anomalous reflections,^[^ ^10^, ^11^ ^]^ cloaking,^[^ ^12^, ^13^, ^14^ ^]^ holography,^[^ ^15^, ^16^, ^17^, ^18^ ^]^ vortex beams generation.^[^ ^19^, ^20^, ^21^ ^]^ Moreover, the metasurface is evolving toward programmable design,^[^ ^22^, ^23^, ^24^ ^]^ adaptive modulation,^[^ ^25^, ^26^, ^27^ ^]^ and intelligent applications,^[^ ^28^, ^29^ ^]^ with the assistance of intelligent algorithms^[^ ^30^ ^]^ and a variety of sensors.^[^ ^31^ ^]^ In particular, space‐time coding metasurfaces demonstrate the capability of beam scanning^[^ ^32^ ^]^ and achieving low scattering.^[^ ^33^, ^34^ ^]^ This is achieved by distributing the energy to the higher harmonics through time modulation, which reduces the radar cross‐section (RCS) at the center frequency. However, these works exhibit high SLLs, primarily due to the absence of simultaneous modulation of amplitude (or gain) and phase. Furthermore, the issue of superposition of harmonics with identical frequency generated by meta‐atoms cannot be fundamentally resolved by solely optimizing space‐time coding. Consequently, it results in a high SBL, which also makes it challenging to achieve further lower scattering. The high SBLs and SLLs have an impact on the transmission of the signal within the target frequency band. It also can be detected by enemy radar, thus exposing the target. Therefore, realizing low SBL and low SLL, particularly ultra‐low SLL with a value of less than −30 dB is extremely critical for covert communication targets. The remarkable scattering modulation capacity of the metasurface has inspired a multitude of research exploring the integration of antennas and metasurfaces for the simultaneous modulation of radiation and low‐scattering characteristics.^[^ ^35^ ^]^ The superposition of a metasurface and antenna array is frequently employed in these studies to reduce the RCS of the entire array while achieving radiation.^[^ ^36^, ^37^, ^38^ ^]^ However, these space‐coding‐only designs cannot realize dynamic beam scanning with low SLLs^[^ ^39^ ^]^ or require a large number of phase shifters and attenuators to accomplish this.^[^ ^40^, ^41^ ^]^ Moreover, most of the works only reduce reflection in the incident direction,^[^ ^39^, ^40^, ^41^ ^]^ and it is challenging to achieve low scattering across the full angular domain.

Recently, some works have begun to explore the integration of metasurfaces and antennas to achieve more functionality concerning radiation scattering.^[^ ^42^, ^43^ ^]^ In a previous publication,^[^ ^44^ ^]^ we proposed an integrated antenna and metasurface design that enables the modulation of radiation gain and the polarization of the reflection EM wave. Some studies have realized reconfigurable radiation‐scattering over the same aperture using only a multilayer cascade structure, including modulation of radiation and in‐band co‐polarized reflection,^[^ ^45^ ^]^ and dynamic control of radiation and in‐band stealth using space‐time‐coding.^[^ ^46^ ^]^ However, the modulation of radiation properties in these studies on radiation scattering remains relatively weak. Despite some radiation‐type metasurfaces achieving 1‐bit,^[^ ^47^ ^]^ 2‐bit, or even 4‐bit^[^ ^48^ ^]^ phase modulation, the ability to achieve arbitrary beam scanning remains elusive. Furthermore, the implementation of only single‐polarization modulation of radiation‐scattering significantly restricts the potential applications of radiation‐scattering type metasurfaces. Therefore, expanding the number of phases and increasing the degrees of freedom to modulate polarization, amplitude, and frequency is imperative for radiation‐scattering type metasurfaces.

**Figure 1:**
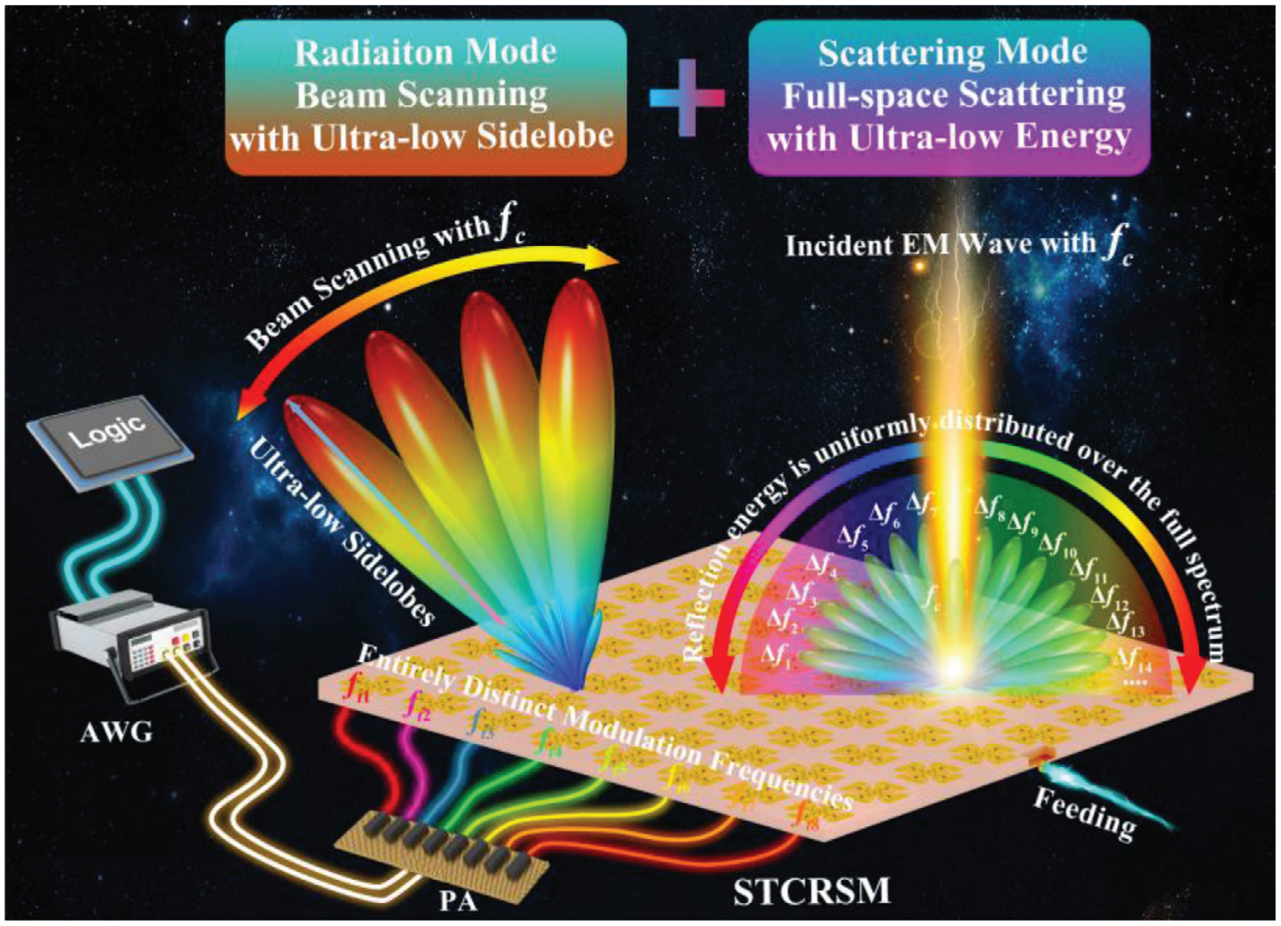
Schematic of the proposed STCRSM. The STCRSM's meta‐atom is capable of independently manipulating the amplitude, phase, polarization, and frequency of radiated and scattered EM waves. With space‐time coding, beam scanning with ultra‐low SLL is achieved by employing nonuniform modulation periods. Ultra‐low in‐band scattering characteristics are also obtained by uniformly distributing the reflection wave energy over the full space and full spectrum.

In this paper, we proposed a space‐time‐coding radiation‐scattering metasurface (STCRSM) for simultaneously modulating amplitude, phase, polarization, and frequency of radiation‐scattering within the same frequency band. An isotropic meta‐atom was designed, comprising four varactor diodes loaded in four directions and a phase‐shifting network (PSN) located at the bottom of the meta‐atom. The modulation of radiation scattering is realized by controlling varactors and PSN. The implementation of the 2‐bit radiation phase and 1‐bit radiation amplitude for both *x*‐ and *y*‐polarization were realized in the radiation mode (RM). The implementation of continuous phase modulation and 1‐bit amplitude modulation is accomplished in the scattering mode (SM). Furthermore, arbitrary amplitude and phase modulation of radiation scattering is realized through time modulation. As illustrated in **Figure** **1**, we have proposed a space‐time modulation method that employs stochastic coding and nonuniform modulation, which entails the operation of each meta‐atom at a distinct modulation frequency. Consequently, beam scanning with ultra‐low SLLs and ultra‐low SBLs, in addition to ultra‐low in‐band scattering, has been realized. The prototype was fabricated and validated in C‐band, and the measurement results were in good agreement with the simulation results.

## 2 Results and Discussion

The schematic diagram illustrating the fundamental principles of the proposed STCRSM is presented in Figure 1. The transition between RM and SM is enabled by controlling the state of the varactor diodes integrated within the meta‐atom. RM represents the modulation of self‐radiation EM waves, which is achieved through the utilization of RF excitation (labeled “Feeding” in Figure 1). The time modulation enables the arbitrary modulation of the frequency, amplitude, and phase of radiation‐scattering. As a demonstration, three typical functions are realized based on a single STCRSM: beam scanning with ultra‐low SLL at center frequency in RM, space‐time modulation for ultra‐low scattering in SM, and beam scanning with in‐band ultra‐low scattering characteristics.

### 2.1 Meta‐Atom Design of STCRSM

#### 2.1.1 Design of the Meta‐Atom Structure

**Figure 2:**
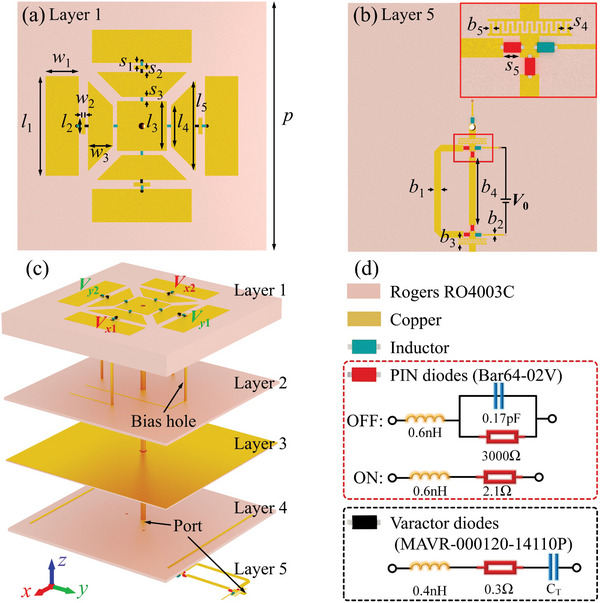
a) The front and b) back layer of the meta‐atom. The geometrical parameters of the meta‐atom are *p* = 30 mm, *l*_1_ = 12 mm, *l*_2_ = 1 mm, *l*_3_ = 6 mm, *l*_4_ = 4.88 mm, *l*_5_ = 10.88 mm, *w*_1_ = 4 mm, *w*_2_ = 0.65 mm, *w*_3_ = 3 mm, *s*_1_ = 0.3 mm, *s*_2_ = 0.15 mm, *s*_3_ = 0.5 mm, *s*_4_ = 0.1 mm, *s*_5_ = 0.7 mm, *b*_1_ = 0.8 mm, *b*_2_ = 0.3 mm, *b*_3_ = 0.7 mm, *b*_4_ = 8.2 mm, *b*_5_ = 0 .1mm. c) 3D schematic of the proposed meta‐atom. d) The supplementary notes of the meta‐atom and the equivalent circuit of the diodes.

In order to achieve a higher degree of integration, a multilayer cascade structure is selected for the simultaneous modulation of radiation and scattering. Accordingly, a modulation layer comprising rectangular and trapezoidal patches is designed, exhibiting absolute symmetry along the *x*‐ and *y*‐axes, as illustrated in **Figure** **2**a. Simultaneously, a phase‐shifting network is integrated into the bottom of the STCRSM structure to acquire more radiation phases, as illustrated in Figure 2b. Four metalized holes connect the outermost four patches of the modulation layer to the feeding layer which are used as the positive terminals of the four varactor bias voltages (*V*_x1_, *V*_x2_, *V*_y1_, and *V*_y2_). The metalized hole in the middle serves both as a common negative terminal for the four voltages and transmits the radiation excitation signal.

The final meta‐atomic structure of the STCRSM is illustrated in Figure 2c and comprises five layers from top to bottom: the modulation layer (layer 1), the varactor diodes feeding layer (layer 2), the metal reflection layer (layer 3), the PIN diodes feeding layer (layer 4), and the PSN layer (layer 5). It is noteworthy that the second and third layers are bonded with a 0.2 mm‐thick layer of Rogers RO4450B, which has a negligible impact on the EM performance of the meta‐atom. The isolation between the DC voltage and the RF signals is primarily dependent on the two interdigital structures and inductors on the bottom of the meta‐atom, as illustrated in Figure 2b. For further details regarding the precise configuration of the feeding network, please refer to Note S1 (Supporting Information). Furthermore, a 1–64 power division network was designed to connect all PSNs, thereby ensuring equal amplitude and in‐phase excitation for all meta‐atoms. For further details on the performance and structure of the power division network, please refer to Note S2 (Supporting Information).

#### 2.1.2 Radiation‐Scattering Performance of the Meta‐Atom

The operating state of the meta‐atom depends on the state of the varactor diode. The constraints of the feeding network result in either a single (RM) or all four (SM) varactor diodes being operational. When all varactor diodes are conducting, the modulation of the reflection phase for arbitrary polarization can be achieved by adjusting the capacitance of the varactor. The phase and polarization of the radiation EM waves can be modulated when only one varactor diode is conducting. The modulation of the *x*‐polarization and *y*‐polarization is achieved by modulating varactor diodes positioned along the *x*‐direction and the *y*‐direction, respectively. To illustrate, the meta‐atom radiates *y*‐polarized waves when a voltage *V*_y1_ is applied, and when a voltage *V*_y2_ is applied, the meta‐atom radiates *y*‐polarized waves but with an intrinsic phase shift of 180°, which is due to the shift obtained by the inversion of the relative spatial position.^[^ ^49^ ^]^ In addition, a 90° phase difference can be introduced by modifying the transmission distance through the control voltage *V*_0_, as shown in Figure 2b. For clarity, the coding states regarding *y*‐polarization are given in **Table** **1**. Furthermore, when all diodes are maintained at the same voltage or no voltage is applied, no radiation is generated at this time. In summary, the meta‐atom is capable of modulating the reflection phase of arbitrary polarization in SM. In RM, the meta‐atom can radiate *x*‐polarized or *y*‐polarized waves with a 2‐bit phase. Furthermore, the meta‐atom is capable of being switched between SM and RM.

**Table 1:** Coding states varying with modulation voltages.

Coding states	*V*_x1_	*V*_x2_	*V*_y1_	*V*_y2_	V_0_	Radiation States
*y*‐1	0	0	+v_1_	0	+v_0_	*y*‐0°
*y*‐2	0	0	+v_1_	0	−v_0_	*y*‐90°
*y*‐3	0	0	0	+v_1_	+v_0_	*y*‐180°
*y*‐4	0	0	0	+v_1_	−v_0_	*y*‐270°
*x*‐1	+v_1_	0	0	0	+v_0_	*x*‐0°
*x*‐2	+v_1_	0	0	0	−v_0_	*x*‐90°
*x*‐3	0	+v_1_	0	0	+v_0_	*x*‐180°
*x*‐4	0	+v_1_	0	0	−v_0_	*x*‐270°
“none”	0	0	0	0	0	nonradiation

**Figure 3:**
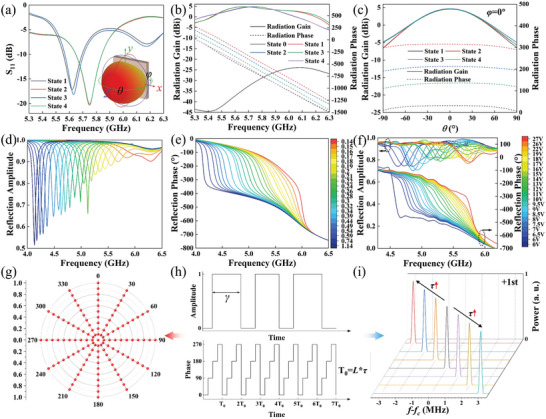
a) The radiation return loss S_11_. b) The radiation gain and phase varying with frequency at *θ* = 0° and *φ* = 0°. c) The radiation gain and phase varying with *θ* when *φ* = 0° and *f*_c_ = 5.7 GHz. The simulated reflection d) amplitude and e) phase varying with frequency and capacities. f) The measured reflection performance of the meta‐atom. g) The arbitrary modulation of amplitude and phase of radiation scattering by time‐coding. h) Schematic diagram of time‐coding. Arbitrary amplitudes can be achieved by controlling the ratio *γ* of radiation (reflection) states. An arbitrary phase can be achieved by controlling the coding of the phase state. i) The time‐coding period can be varied by changing the value of *τ*, thereby achieving arbitrary frequency shifts through the modulation of the ±1st harmonics.

A series of rigorous modeling and simulations are conducted in CST microwave studio to verify the radiation‐scattering performance of the meta‐atom. The varactor diode and switching diode are set up as equivalent circuits, as shown in Figure 2d. In RM, the four radiation states of S_11_ and the broadband radiation gain and phase indicate that the meta‐atom can realize good radiation with a 2‐bit phase. Additionally, the radiation patterns shown in Figure 3c indicate that a constant phase difference can be maintained across a broad angular range. The gains of the four coding states are kept identical across a broad angular range. The modulation of radiation performance is primarily accomplished through the modulation of surface current, as detailed in Note S3 (Supporting Information). As shown in Figure 7j,k, the radiation performance of the prototype was measured when all the meta‐atoms were maintained in the same state, which also demonstrated that the meta‐atoms exhibited a stable phase difference. The aperture efficiency at 5.7 GHz is ≈30% for equal amplitude in‐phase excitation, which can be optimized by optimizing the power division and DC feeding network. In SM, broadband tunable reflection phases were obtained, with the reflection amplitudes typically greater than 0.8. The measured reflection amplitude and phase are shown in Figure 3f, which are in good agreement with the simulation results. Four states were selected for space‐time‐coding: 0 V (−578°), 16 V (−485°), 22 V (−398°), and 26.8 V (−307°). It should be noted that the radiation and scattering performances are identical for *x*‐polarization and *y*‐polarization since the proposed meta‐atom is entirely symmetric with regard to the *x*‐axis and *y*‐axis. Hence, only the radiation performance of *y*‐polarization is given in **Figure** **3**. The radiation and scattering performances of *x*‐polarization are given in Note S4 (Supporting Information). Subsequently, *y*‐polarization is employed as a demonstration to validate the proposed strategy.

### 2.2 Modulation Theory and Methodology

#### 2.2.1 Space‐Time‐Coding Theory

The method of modulating the radiation performance by time coding is frequently designated as a time‐modulated antenna (TMA).^[^ ^50^, ^51^ ^]^ Both time‐modulated metasurfaces (TMMs) and TMAs are inherently coding the tunable states of the meta‐atom (antenna) in the time dimension.^[^ ^33^ ^]^ Consequently, frequency modulation can be achieved by coding tunable states. For a time‐modulated array comprising M*N meta‐atoms, the far‐field at center frequency can be expressed as follows:

(1) $$\begin{array}{cc} f(\theta ,\varphi ,t) & =\sum_{q=1}^{N}\sum_{p=1}^{M}E_{pq}\left(\theta ,\varphi \right)\Gamma_{pq}\left(t\right)\\ & \;\times \exp\left\{jk\sin\theta [(p-1)d\cos\varphi +(q-1)d\sin\varphi ]\right\} \end{array}$$

where *k* = 2π/*λ*_c_ is the wavenumber and *λ*_c_ is the wavelength of center frequency *f*_c_, *d* denotes the distance between two meta‐atoms. *θ* and *φ* represent the elevation and azimuth angle, respectively. *E*_pq_(*θ*, *φ*) represents the far‐field pattern of the (*p*, *q*) meta‐atom at the center frequency *f*_c_. Γ_pq_(*t*) is the time‐modulated radiation (reflection) coefficient of the meta‐atom, which is a periodic function of time, i.e., Γ_pq_(*t*) = Γ_pq_(*t+T*_0_). During a modulation period *T*_0_, Γ_pq_(*t*) consists of *L* rectangular pulse functions.

(2) $$\Gamma_{pq}\left(t\right)=\sum_{n=1}^{L}a_{pq}^{n}e^{j\beta_{pq}^{n}}U_{pq}^{n}\left(t\right)\left(0<t<T_{0}\right)$$

(3) $$U_{pq}^{n}\left(t\right)=\left\{\begin{array}{cc} 1 & (n-1)\tau \leq t<n\tau \\ 0 & Others \end{array}\right.$$

where $\alpha_{pq}^{n}$ and $\beta_{pq}^{n}$ are the tunable amplitude and phase of the (*p*, *q*) meta‐atom. *τ* = *T*_0_/*L* is the width of the rectangular pulse. For the sake of simplicity in presentation, all subsequent symbols will be presented without the subscript “*pq*.” Decomposing *U^n^* (*t*) into Fourier series, the Fourier series coefficient *r^m^* of Γ(*t*) can be obtained.

(4) $$r^{m}=\sum_{n=1}^{L}a^{n}\frac{\sin(\pi m/L)}{\pi m}\exp\left(j\beta^{n}-j\pi m\left(2n-1\right)/L\right),\;m\neq 0$$

(5) $$r^{0}=\sum_{n=1}^{L}\frac{a^{n}\exp\left(j\beta^{n}\right)}{L}$$

where *m* denotes the harmonic order, *f*_m_ = *f*_c_ + *mf*_0_, *f*_0_ = 1/*T*_0_ is the switching frequency.

#### 2.2.2 Independent Modulation of Amplitude, Phase, Polarization, and Frequency of Radiation‐Scattering

The modulation of the radiation amplitude and phase by the meta‐atom, as well as the modulation of the reflection phase, have been discussed in detail in Section 2.1.2. It is worth mentioning that when the meta‐atom is in RM, the reflection amplitude is 0 since the incident EM wave is completely received. Accordingly, there are five coding states for RM and SM, whereby two states (0, 1) for *a^n^* and four states (2‐bit) for *β^n^*. For polarization modulation, the switching of radiating *x*‐polarized and *y*‐polarized EM waves can be achieved by switching the voltage in RM. The reflection phase modulation for arbitrary polarization can be achieved in SM.

Considering *β^n^* = 0, the resolution of the equivalent amplitude obtained by amplitude coding is 1/*L* according to Equation (5). Similarly, phase coding enables the implementation of arbitrary phases of radiation scattering, as shown in Figure 3g. According to Equation (4), the modulation of frequency can be achieved through the modulation of the ±1st harmonic, with a frequency shift of ±1/(L*τ). The +(−)1st harmonic is generated when the phase is increasing (decreasing) on the time sequences, as shown in Figure 3h. Arbitrary frequency shifts can be achieved by changing L*τ. The frequency shift is inversely proportional to the value of *τ*, as shown in Figure 3i. Furthermore, coding states with a greater number of bits enable more energy to be concentrated into the ±1st harmonic,^[^ ^51^ ^]^ as elucidated in Note S5 (Supporting Information). The phase modulation of the +1st harmonic can be achieved by altering the order of the time‐coding sequence without affecting its amplitude.

#### 2.2.3 Principle of Beam Scanning with Ultra‐Low SLL

The principal objective of time coding is to achieve equivalent amplitude, phase, and frequency modulation. The modulation efficiency depends on the time‐coding sequences and the tunable state of the meta‐atom. Consider obtaining the equivalent amplitude and phase of the center frequency. According to Equation (5), the reflection coefficients are time‐weighted and independent of the coding order, so the target phase *β* must satisfy the following condition:

(6) $$\beta^{n,1}\leq \beta <\beta^{n,2},\;\beta^{n,2}=\pi /2+\beta^{n,1}$$

where *β^n,^* ^1^ and *β^n,^* ^2^ are two tunable states of the meta‐atom. Given that these tunable states have equal amplitudes, the quantities of *β^n,^* ^1^ and *β^n,^* ^2^ can be ascertained as o_1_ and o_2_ when *L* is determined. The amplitude of the equivalent phase is:

(7) $$a=\sqrt{{o_{1}}^{2}+{o_{2}}^{2}}/L,\;o_{1}+o_{2}=L$$

When the target phase differs, its corresponding equivalent amplitude also differs. In order to achieve low‐sidelobe beam scanning, the amplitude distribution of the array should satisfy the Taylor distribution. Here, the amplitude weighting is achieved by introducing states with amplitude 0. Hence, the final coding states are 0, exp(*β^n,^* ^1^), exp(*β^n,^* ^2^) corresponding to quantities o_0_, o_1_ and o_2_, respectively. The equivalent radiation coefficient at the center frequency can be expressed as:

(8) $$r^{0}=\left(o_{1}*\exp\left(\beta^{n,1}\right)+o_{2}*\exp\left(\beta^{n,2}\right)\right)/L$$

Accordingly, arbitrary amplitudes and phases can be obtained. However, Equation (4) indicates that time‐coding generates harmonics, which highly correlate with the order of coding. It is essential to suppress the sideband levels (SBLs) to prevent harmonics from affecting the transmission of the center frequency signal. Here, it is assumed that the frequency of the *m*_q1_‐th order harmonic of *q*_1_‐th meta‐atom has the same frequency as the *m*_q2_‐th order harmonic of *q*_2_‐th meta‐atom. In such a scenario, we can then conclude that:

(9) $$\begin{array}{cc} f_{\mathrm{equal}} & =f_{c}+m_{1}/\left(L*\tau_{1}\right)=f_{c}+m_{2}/\left(L*\tau_{2}\right)\\ & =\cdot \cdot \cdot =f_{c}+m_{8}/\left(L*\tau_{8}\right) \end{array}$$

(10) $$f_{\mathrm{equal}}-f_{c}=\frac{m_{1}}{L*\tau_{1}}=\frac{m_{2}}{L*\tau_{2}}=\cdot \cdot \cdot =\frac{m_{q}}{L*\tau_{q}}$$

Each meta‐atom generates a large number of harmonics with the same frequency, which are superimposed in space, thereby resulting in greater energy. However, the harmonic amplitude of a single meta‐atom is inversely proportional to the harmonic order according to Equation (4). Hence, the key to achieving SBL suppression is to ensure that the first same frequency appears at higher‐order harmonics. This can be achieved by ensuring that Min(*m*_q_) is as large as possible. If the same *τ*_q_ is used for each meta‐atom, all the meta‐atoms will have the same frequency at ±1st harmonics, which generates a high energy. Assuming that *τ*_q_ are all integers and the common divisor of *τ*_q_ is *τ*_0_, then the corresponding harmonic orders of each meta‐atom with the same frequency is *τ*_q_/*τ*_0_. Therefore, *τ*_q_ should be as large as possible and it is necessary to ensure that *τ*_0_ = 1 to suppress harmonics efficiently. Hence, we set *τ*_q_ = (1.09+q*0.1) *us*, the order in which the first meta‐atom appears to have harmonics with the same frequency is the 119th order. Moreover, stochastic coding is employed to distribute harmonics more uniformly across the entire spectrum once the number of coding states has been determined. In conclusion, stochastic coding and nonuniform modulation are utilized to achieve a lower SLL. In comparison to sequential coding, stochastic coding reduces the SBL by ≈17.78 dB, and nonuniform modulation can reduce the SBL by ≈8.8 dB. As *L* increases, lower SBLs can be achieved due to the fact that larger *L* distributes the harmonic energy more uniformly over the spectrum according to Equation (4). Additionally, the implementation of different SLLs has a relatively minimal effect on the SBL. A more detailed discussion can be found in Note S6 (Supporting Information). Furthermore, the larger *L* achieves a more precise equivalent amplitude and phase at the center frequency according to Equation (8). Nevertheless, the error in the equivalent magnitude and phase obtained when *L* exceeds 1000 is already minimal, as demonstrated in Note S7 (Supporting Information). However, an *L* that is too large results in a more complex modulation and an increased modulation error. Consequently, in light of the impact of *L* on SBL, *L* = 1000 has been selected as the validation value.

### 2.3 Manipulation of Radiation‐Scattering

#### 2.3.1 Radiation Beam Scanning with Ultra‐Low SLL at Center Frequency

**Figure 4:**
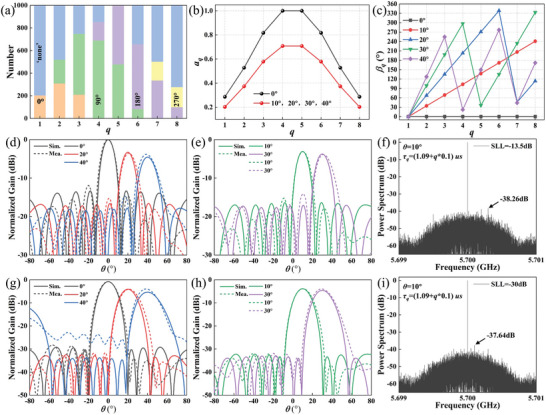
a) The number of coding states in the time‐coding sequence of each meta‐atom when *θ* = 10° and SLL = −30 dB. The b) amplitude and c) phase distributions are required for beam scanning with SLL = −30 dB. The simulated and measured results of beam scanning when d,e) SLL = −13.5 dB and g,h) SLL = −30 dB. f,i) The normalized power spectrum measured for different SLLs. A reduction in SLL and an increase in *θ* result in a corresponding decrease in radiation gain. The variation in SLL has a negligible impact on SBL.

Next, the one‐dimensional beam scanning was verified. The relationship between the ideal phase gradient and the angle of the beam scanning is as follows:

(11) $$\begin{array}{c} \Delta \beta_{x}=-\left(q-1\right)kd\sin\left(\theta \right)\cos\left(\varphi \right)\\ \Delta \beta_{y}=-\left(q-1\right)kd\sin\left(\theta \right)\sin\left(\varphi \right) \end{array}$$

where Δ*β*_x_ (Δ*β*_y_) represents the phase difference in the *x*‐ (*y*‐) direction. The case of a one‐dimensional beam scan with *φ* = 0° and *θ* = 0°, 10°, 20°, 30°, 40° is considered here. Here, *L* = 1000 and SLL = −13.5, and −30 dB were chosen for validation. As a consequence of time modulation, the maximum equivalent amplitude at scanning angles of *θ* = 10°, 20°, 30°, and 40° is 0.707 at the center frequency. However, the maximum equivalent amplitude can be 1 for *θ* = 0°, as no phase gradient is required. The required amplitude and phase of each meta‐atom for beam scanning with ultra‐low SLL (−30 dB) are given in **Figure** **4**b,c. For SLL = −13.5 dB, the phase distribution is the same as for SLL = −30 dB, while the amplitude is identical for all meta‐atoms.

The proportion of each coding state in the time‐coding sequence for each meta‐atom was calculated by Equation (8), as illustrated in Figure 4a. Subsequently, the time‐coding sequence for each meta‐atom can be obtained through stochastic coding of the coding states. The simulated and measured radiation patterns are shown in Figure 4. It should be noted that the maximum gain (*θ* = 0° and SLL = −13.5 dB) is used as the normalizing factor for all radiation patterns. Indeed, a case of *θ* = 0° and SLL = −13.5 dB represents an equal amplitude and in‐phase excitation with no time modulation. The gain for *θ* = 0° is ≈3.5 dB greater than other scanning angles for an identical SLL, which is predominantly due to the time‐coding equivalent amplitude, as shown in Figure 4b. However, the time modulation loss is outweighed by the considerable savings in phase shifters and attenuators, which can be compensated for by increasing the input power. In addition, the gain for SLL = −30 dB is ≈1 dB smaller than the gain for SLL = −13.5 dB, primarily due to the fact that the required amplitude distribution for the beam scanning with low SLL introduces a greater number of “none” states in time‐coding sequences. Hence, the gain loss essentially arises from time modulation, which encompasses the influences of both scanning angle and SLL. As the SLL decreases, the increasing number of “none” states results in an increase in gain loss and a concomitant decrease in power efficiency. More detailed discussion can be found in Note S8 (Supporting Information). For SLL = −13.5 dB, the actual measured SLL is −12, −12.1, −11.7, −10.2, and −10.9 dB at scanning angles *θ* = 0°, 10°, 20°, 30°, and 40°, respectively. For SLL = −30 dB, the actual measured SLL is −27.5, −28.2, −27.4, and −27.9 dB at scanning angles *θ* = 0°, 10°, 20°, and 30°, respectively. The measured SLL is ≈3 dB greater than the ideal SLL, which is within the experimental error range. This discrepancy may be attributed to the fact that the amplitudes of the coding states are not strictly identical, resulting in a deviation from the Taylor distribution. However, the deterioration in performance is evident in the significant gain lift from −80° to 0°, with a sharp increase in sidelobe level when SLL = −30 dB and *θ* = 40°. The observed degradation in performance can be attributed to two primary factors. The first is the limited number of meta‐atoms in the array, and the second is the large period of the meta‐atom. This can be improved by increasing the number of meta‐atoms and optimizing the period of the meta‐atom, which is demonstrated in Note S9 (Supporting Information). The measured spectra indicated that the maximum SBLs were −38.26 and −37.64 dB, respectively, for SLL = −13.5 and SLL = −30 dB. These values are slightly higher than those obtained from the simulations presented in Note S6 (Supporting Information). In any case, the proposed strategy effectively suppresses the SBL. Overall, the measurements coincide very well with the simulations, but there are some discrepancies in the scanning angle and gain. These discrepancies are present in both the array and the meta‐atom, and may be attributed to the following factors: 1) Since the simulation of the meta‐atom uses periodic boundary conditions, which are not satisfied when the radiation pattern modulation is performed; 2) The coupling between the array elements; 3) The loss introduced by the fifth layer of the power‐division network, which was not incorporated into the simulation of the meta‐atom; 4) Errors from fabrication, soldering, etc. As the scanning angle is increased, the widening of the radiation beam results in a broader distribution of radiant energy in each direction, leading to an increased SLL. Furthermore, the proposed strategy enables the realization of beam scanning with lower SLLs. However, this has yet to be experimentally verified due to the limitations of the measurement conditions, as detailed in Note S10 (Supporting Information).

#### 2.3.2 Space‐Time Modulation for Ultra‐Low Scattering

For scattering modulation, the ability of STCRSM to modulate the reflected beam is demonstrated. Nevertheless, the deflection of the incident EM wave into the nonincident direction is extremely unreliable. This is because that energy deflected into other angular domains may also be detected. Here, the reflection in the full‐angle domain can be reduced by employing space‐time modulation to distribute the incident EM wave energy uniformly to all order harmonics. In the following, the demonstration will be based on the *y*‐polarizations, given that the meta‐atom is isotropic and exhibits a consistent response to arbitrary polarizations.

**Figure 5:**
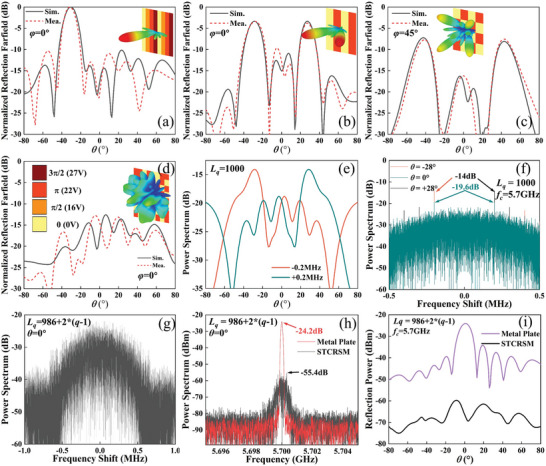
The simulated and measured far‐field patterns using space‐only coding for a) single‐beam deflection, b) two‐beam deflection, c) four‐beam deflection, and d) random distribution. e) The simulated power patterns with *θ* for two harmonics with a significantly higher power. f) The simulated power patterns with Δ*f* for two angles with significantly higher power. g) The simulated reflection power spectrum with optimized *L*_q_. h) The measured power spectrum of metal plate and STCRSM. i) The reflection power pattern with *θ* of the metal plate and STCRSM. The optimized *L*_q_ reduces the power of power‐increasing harmonics and the full space scattering is substantially reduced compared to the metal plate.

For space coding to reduce echo in the incident direction, two principal methods may be employed. The first is to reduce the echo by deflection, while the second is to distribute the echo energy as uniformly as possible throughout the space by random coding. Nevertheless, there will be particular angles that exhibit greater energy for both methods. Here, four different space codes are designed to demonstrate the STCRSM's ability to modulate the beam. The four space codes modulate the reflection beams into the following distributions: single‐beam deflection, two‐beam deflection, four‐beam deflection, and random distributions. In the case of single‐beam deflections, the energy is concentrated at (−30°, 0°), which implies that *θ* = −30° and *φ* = 0°. This reduces the echo in the incident direction but dramatically increases the energy at a specific angle (−30°, 0°). In the case of two‐beam and four‐beam deflection, this implies that the energy is distributed uniformly across multiple directions. However, the reflected energy remains large at specific angles. Furthermore, it is challenging to achieve a uniform distribution of energy in space for random codes, as there still exists a large energy in specific angles. The four space codes were simulated in CST and the results are shown in **Figure** **5**a–d. All reflection far‐field patterns are normalized to the maximum value of the single‐beam deflection energy. At *θ* = 0°, the reflection energies for single‐beam deflection, double‐beam deflection, quadruple‐beam deflection, and uniform distribution are −16.9, −15, −22, and −20.4 dB, respectively. However, their maximum reflection energies in the whole space are 0, −3.3, −7.3, and −12.5 dB, respectively. It is evident that the energy in the other angular domains would be significantly larger, which would also have an extremely detrimental impact on the target's stealth performance.

A space‐time‐coding strategy will be employed next to achieve ultra‐low scattering characteristics across the full angular domain and the full operating frequency band. In addition to the uniform distribution of energy in space, space‐time modulation can also achieve a more uniform distribution in frequency, which significantly reduces the maximum reflection energy. In RM, stochastic coding and nonuniform modulation are employed to reduce the SBL of beam scanning. When Equation (10) is satisfied, all the meta‐atoms generate a harmonic of the same frequency *f*_equal_, which increases reflection energy. Nevertheless, given that the individual meta‐atoms will exhibit disparate equivalent phases at *f*_equal_, the angle at which the maximum energy lies will not be the same as the scanning angle. Rather, it will be indefinite. Hence, there will be no effect on beam scanning.

In this example, 0 and π coding state is employed for illustrative purposes. The number of states in the time‐coding sequence must be equal to reduce the reflection at the center frequency. Note S11 (Supporting Information) illustrates that the number of coding states has a negligible impact on reflection reduction. Consider *τ*_q_ = (1.09+q*0.1) *us*, the order in which the first meta‐atom appears to have harmonics of the same frequency is the 119th order, which is wholly contingent upon *τ*_1_. The corresponding value of Δ*f* is equal to ±0.1*N MHz (*N* = 1, 2, 3, …) when *L* = 1000. The simulation results are shown in Figure 5e,f. It is evident that the reflection energy increases significantly at Δ*f* = ±0.2 MHz, reaching a 5.6 dB higher than the second highest energy. Furthermore, the reflection pattern at Δ*f* = ±0.2 MHz indicates a considerable increase in energy over a broad angular range. Indeed, if the value of *τ*_1_ is sufficiently finely sampled, for instance, *τ*_1_ = 1.1009 µs enables *f*_equal_ to emerge at a higher order, thereby reducing the peak values. Unfortunately, the limitations of the control equipment make it impossible to achieve such subtle modulation of the *τ*_q_.

(12) $$f_{0}=\frac{1}{T_{0}}=\frac{m_{1}}{L_{1}*\tau_{1}}=\frac{m_{2}}{L_{2}*\tau_{2}}\cdot \cdot \cdot \cdot =\frac{m_{q}}{L_{q}*\tau_{q}}$$

In light of the modulation constraints of *τ*, it is necessary to give further consideration to each meta‐atom with varying *L*. In order to guarantee that the reflection energy at the center frequency is zero, it is necessary to calculate the value of *L* as follows: *L*_q_ = *L*_0_ + (*q*‐1)*k*b, where *b* denotes the number of coding states, *L*_0_ is a constant that is an integer multiple of *b*, *k* is an integer greater than 1. Here, take *L*_0_ = 986, *b* = 2 and *k* = 1. The order in which the first meta‐atom appears to have harmonics of the same frequency is 58667th order with Δ*f* = ±0.1 GHz. Due to the limitation of computational resources, only the spectrum in the range Δ*f* = ±1 MHz is calculated. As illustrated in the Figure 5g, the maximum reflection energy is −21.87 dB. The reflection pattern could not be calculated because the same frequency did not appear in the calculated frequency band. It is evident that the incorporation of nonuniform *L* markedly suppresses the reflection energy. The reflection power spectrum of STCRSM with varying *L* was measured as shown in Figure 5h. The proposed strategy achieves a 31.2 dB reduction at *θ* = 0° in comparison to the reflection power spectrum of the metal plate. Figure 5i also demonstrates that the proposed strategy achieves ultra‐low scattering across the entire angular domain.

#### 2.3.3 Beam Scanning with In‐Band Ultra‐Low Scattering Characteristics

**Figure 6:**
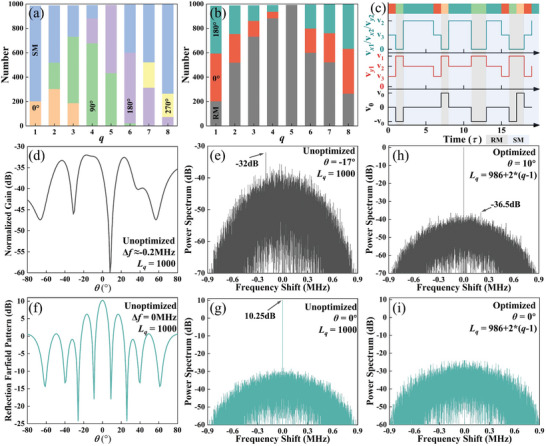
a) Number of coding states for each meta‐atom used for beam scanning with *θ* = 10° and SLL = −30dB. b) The optimized time‐coding sequences for in‐band ultra‐low scattering characteristics. c) The initial 20 bits of the time‐coding sequence of the second meta‐atom and the corresponding modulation voltages. d‐g) The simulated performance with the unoptimized space‐time‐coding sequence: There is a surge in harmonic energy at Δ*f* = −0.2 MHz: d) the radiation pattern and e) the radiation power spectrum with a significantly higher power. There is a strong reflection power at *θ* = 0°: f) The reflection pattern and h) the power spectrum. h, i) The simulated performance with the optimized space‐time‐coding sequence: h) The radiation power spectrum, and i) the reflection power spectrum. The optimization has no impact on radiation performance but greatly reduces reflections.

As illustrated in Figure 4a, beam scanning with low SLL requires a considerable number of “none” states in the time‐coding sequence, namely the meta‐atom maintains the SM. It will undoubtedly expose the target when confronted with the detection of enemy radar operating at the same frequency as the radiation. By optimizing *L*_q_ for each meta‐atom and coding the “none” state in the time‐coding sequence, it is possible to achieve beam scanning with ultra‐low SLL as well as in‐band ultra‐low scattering characteristics. As previously outlined in subsection 2.3.2, the application of a uniform *L* value leads to an increase in energy within specific angular domains when operating beam scanning. The beam scanning of the simulation and measurement, conducted with the identical *L*, are already presented in Figure 4. The maximum SBL of −32 dB outside the scanning angle was found by simulation, as shown in Figure 6d,e. This value is observed at *θ* = −17° and Δ*f* ≈ −0 .2MHz. It should be noted that this is only the result for a specific space‐time‐coding sequence, which varies for different space‐time‐coding sequences. Consequently, it can be concluded that *L*_q_ = 986 + (*q*‐1)*2 and *τ*_q_ = (1.09 + q*0.1) *us* are the optimal choice. Without loss of generality, the radiation‐scattering modulation capability of STCRSM is verified with *θ* = 10° and SLL = −30dB.

**Figure 7:**
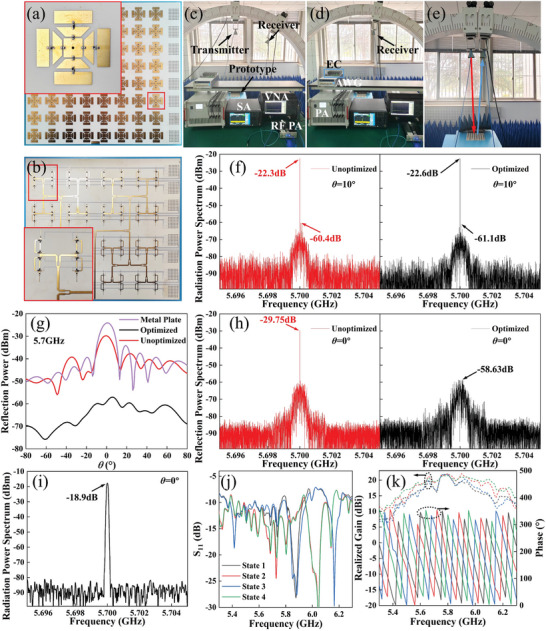
The a) front and b) back of the fabricated prototype. Measurement setups for c) radiation performance, d) scattering performance, and e) meta‐atom performance. The measured f) radiation spectrum and h) reflection spectrum with unoptimized and optimized space‐time‐coding sequence. g) The measured reflection power varying with *θ*. i) The measured radiation spectrum when all meta‐atoms are in state 1. The measured j) S_11_, k) realized gain and phase of four states when all meta‐atoms are in the same state.

**Figure** **6**a illustrates the number of each coding state obtained using nonuniform *L* values for each meta‐atom. The minor alteration of *L* has no impact on the beam scanning at the center frequency, as shown in Figure 6h. When all varactor diodes of the meta‐atom maintain the same capacitance, it is in a nonradiation state, which corresponds to a state of SM. If the same SM is maintained, the reflection energy will be concentrated at the center frequency, which will be significantly elevated. As illustrated in Figure 6g, the peak value is 10.25 dB. Furthermore, the reflection pattern at the center frequency indicates that the reflection energy is large over a wide angular range. As illustrated in Figure 6b, the SM states in the original time‐coding sequence were stochastically assigned equal proportions of 0° and 180° phases to reduce the reflection energy. The initial 20 bits of the time‐coding sequence of the second meta‐atom and corresponding voltage modulation sequences are illustrated in Figure 6c. In this context, the modulation sequences of V_y2_, V_x1,_ and V_x2_ are identical, as the time‐coding sequence employed for modulating the *y*‐polarized EM wave radiation exclusively utilizes the 0° and 90° radiation phase states. Figure 6i illustrates the power spectrum of the optimized scattering code at 0°. Although the energy is increased at all other harmonics, the maximum peak is reduced to −23.9 dB. The radiation‐scattering performance was measured using both an unoptimized and an optimized space‐time‐coding sequence. As shown in Figure 7f–h, the radiation performance remains unaffected, while the reflection is reduced across the full angular domain, particularly at 0° by 28.88 dB in comparison to the unoptimized sequence.

## 3 Conclusion

To summarize, we proposed a strategy for realizing beam scanning with ultra‐low SLL and in‐band ultra‐low scattering characteristics. The proposed STCRSM is subtly designed with an isotropic meta‐atom, in which the switching between RM, SM, and radiation‐scattering mode is achieved by modulating four integrated diodes. A space‐time coding method with nonuniform modulation and stochastic coding is proposed. Dynamic beam scanning with ultra‐low SLL is achieved by co‐coding the amplitude and phase while suppressing the SBL. Ultra‐low scattering is achieved in the full spectrum over the angular domain by uniformly spreading the energy to the harmonics. A prototype was fabricated and the space‐time modulation capability of STCRSM was verified in the C‐band. The scanning angles of 40°, 30°, and 20° were verified when the SLL was equal to −13.5, −30, and −40 dB, respectively. Furthermore, out‐of‐band stealth can be achieved by combining frequency‐selective absorption surfaces when both radiation modulation and in‐band stealth are accomplished. The proposed STCRSM facilitates a high degree of integration of multiple EM functions and markedly reduces the number of apertures of the platform. The advantage of the robust capacity to arbitrarily modulate the amplitude and phase endow it with considerable potential in covert communication systems, radar systems, and compact aerospace equipment.

## 4 Experimental Section

### Simulations

The simulation of the radiation and scattering performance of the meta‐atom was conducted using the commercial EM simulation software CST Microwave Studio, based on the finite element method for solving the full 3D Maxwell equations. For the scattering performance of the meta‐atom, the boundary conditions are periodic in the *x‐* and *y‐*directions and open in the *z‐*direction, with the linear polarized EM wave incident along the +*z*‐direction. For the radiation performance of the meta‐atom, the boundary conditions are open in the *x*‐direction, *y*‐direction, and *z*‐direction. All time‐coding sequences and simulated radiation patterns were generated through the utilization of MATLAB calculations.

### Measurements

All measurements were performed in an anechoic chamber. The modulation speed of the eight array elements was set to *τ*_q_ = (1+0.09*q) *us*, *L*_q_ = 986 + (*q*‐1)*2 (*q* = 1, 2, …, 8), and SLL = −13.5 and −30 dB. Subsequently, the time‐coding sequences for beam scanning at 0°, 10°, 20°, 30°, and 40° are generated in accordance with the methodology outlined in Section 2.2.3. The square wave signal generated by the arbitrary signal generator (AWG) is passed through the power amplifier (PA) and expansion circuitry (EC), which in turn generates the control signal. The PA uses the ATA‐D60090 produced by Aigtek. In order to calibrate the device for radiation measurement, a standard gain antenna is measured. Similarly, a metal plate of the same dimensions is measured in order to calibrate the device for reflection performance.

When measuring the radiation patterns, the prototype and receiving antennas are connected to an Agilent Vector Network Analyzer (VNA) E8363B. The excitation of the prototype was set to 5.7 GHz. The RF power consumption with an RF power amplifier is ≈13 dBm. The receiving antenna moves along a slide to receive and analyze radiation EM waves at intervals of 1°, as shown in Figure 7d. When measuring the power spectrum, the scanning angle *θ* was set at 10° in order to validate the SBL suppression. The angle between the receiving antenna and the prototype normal has been set to 10°, and the receiving antenna is connected to the spectrum analyzer (SA) N9000B. The prototype was connected to a VNA, with excitation frequencies set to 5.7 GHz. The radiation EM waves from the prototype were analyzed by SA.

When measuring the reflection performance of the meta‐atom, the identical voltage is maintained for all meta‐atoms. The transmitting antenna and receiving antenna are slightly angled for vertical incidence, as shown in Figure 7c. For measuring the scattering performance, the transmitting and receiving antennas were connected to the VNA and SA, as shown in **Figure** **7**a.

## Conflict of Interest

The authors declare no conflict of interest.

## Supporting information



Supporting Information

## Data Availability

Data sharing is not applicable to this article as no new data were created or analyzed in this study.
