# Supplementary material for: Beam Scanning with Ultra‐Low Sidelobes and In‐Band Ultra‐Low Scattering Characteristics Empowered by Single Space‐Time‐Coding Radiation‐Scattering Metasurface
Source: Adv Sci (Weinh). 2025 Jan 29;12(11):2413429. doi: 10.1002/advs.202413429 (PMC11923893; doi:10.1002/advs.202413429)
Supplement: Supplementary file 1 — Supporting Information [file ADVS-12-2413429-s001.pdf]

ADVANCED SCIENCE
Open Access

Supporting Information

for *Adv. Sci.*, DOI 10.1002/advs.202413429

Beam Scanning with Ultra-Low Sidelobes and In-Band Ultra-Low Scattering Characteristics Empowered by Single Space-Time-Coding Radiation-Scattering Metasurface

Lixin Jiang, Yongfeng Li, Hao Yang, Mingbao Yan, Jinming Jiang, Yunwei Zhang, Zhe Qin, Wanwan Yang, Hongya Chen, Yongqiang Pang, Zhihao Guo, Lin Zheng, Jiafu Wang and Shaobo Qu*

Supporting Information for "Beam Scanning with Ultra-Low Sidelobes and In-band Ultra-Low Scattering Characteristics Empowered by Single Space-Time-Coding Radiation-Scattering Metasurface"

Lixin Jiang, Yongfeng Li*, Hao Yang, Mingbao Yan, Jinming Jiang, Yunwei Zhang, Zhe Qin, Wanwan Yang, Hongya Chen, Yongqiang Pang, Zhihao Guo, Lin Zheng, Jiafu Wang, and Shaobo Qu

L. Jiang, Prof. Y. Li, H. Yang, Prof. M. Yan, Prof. J. Jiang, Y. Zhang, Z. Qin, W. Yang, Prof. H, Chen, Z. Guo, Prof. L. Zheng, Prof. J. Wang, and Prof. S. Qu

Shaanxi Key Laboratory of Artificially-structured Functional Materials and Devices. Airforce Engineering University, Xi'an, Shaanxi 710051, China

Prof. Y. Pang

Electronic Materials Research Laboratory Key Laboratory of Ministry of Education Xi'an Jiao-tong University Xi'an, Shaanxi 710049, China

E-mail: liyf217130@126.com

Keyword: radiaton-scattering; space-time-coding metasurface

This supporting information includes:

Supplementary Notes 11

Figures 11

Note 1. The detailed structure of the feeding network

The width of all feeding lines is 0.15mm, with a spacing of 0.1 mm between them. The radius of the rightmost hole of the prototype is 0.4mm, with a distance of 2.54 mm between the centers of the apertures.

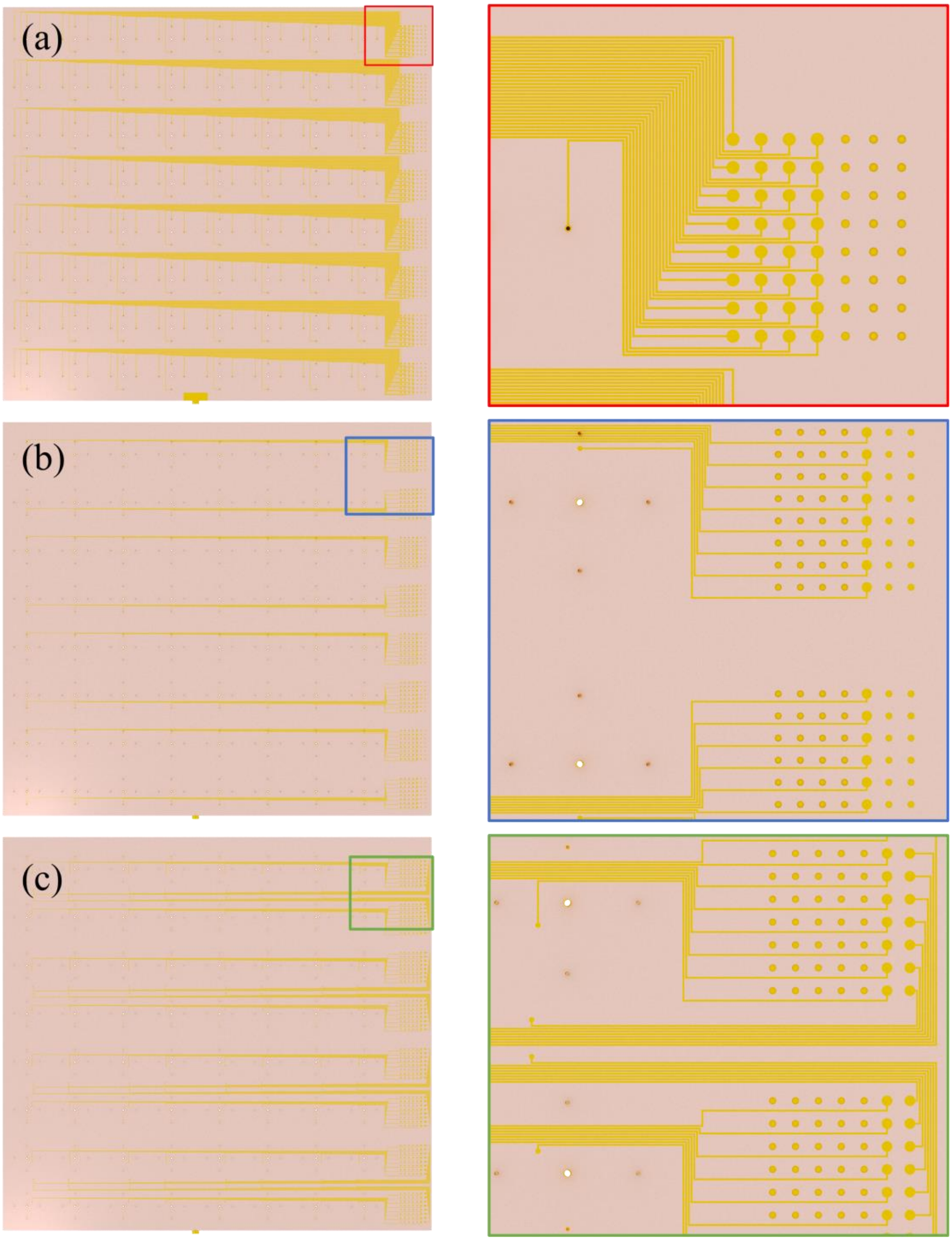

Figure S1. a) The feeding networks for voltages V_{x1}, V_{x2}, V_{y1}, and V_{y2}. b) The feeding networks for common negative. c) The feeding networks for the phase-shifted network (PSN).

Note 2. The detailed structure and performance of the power division network

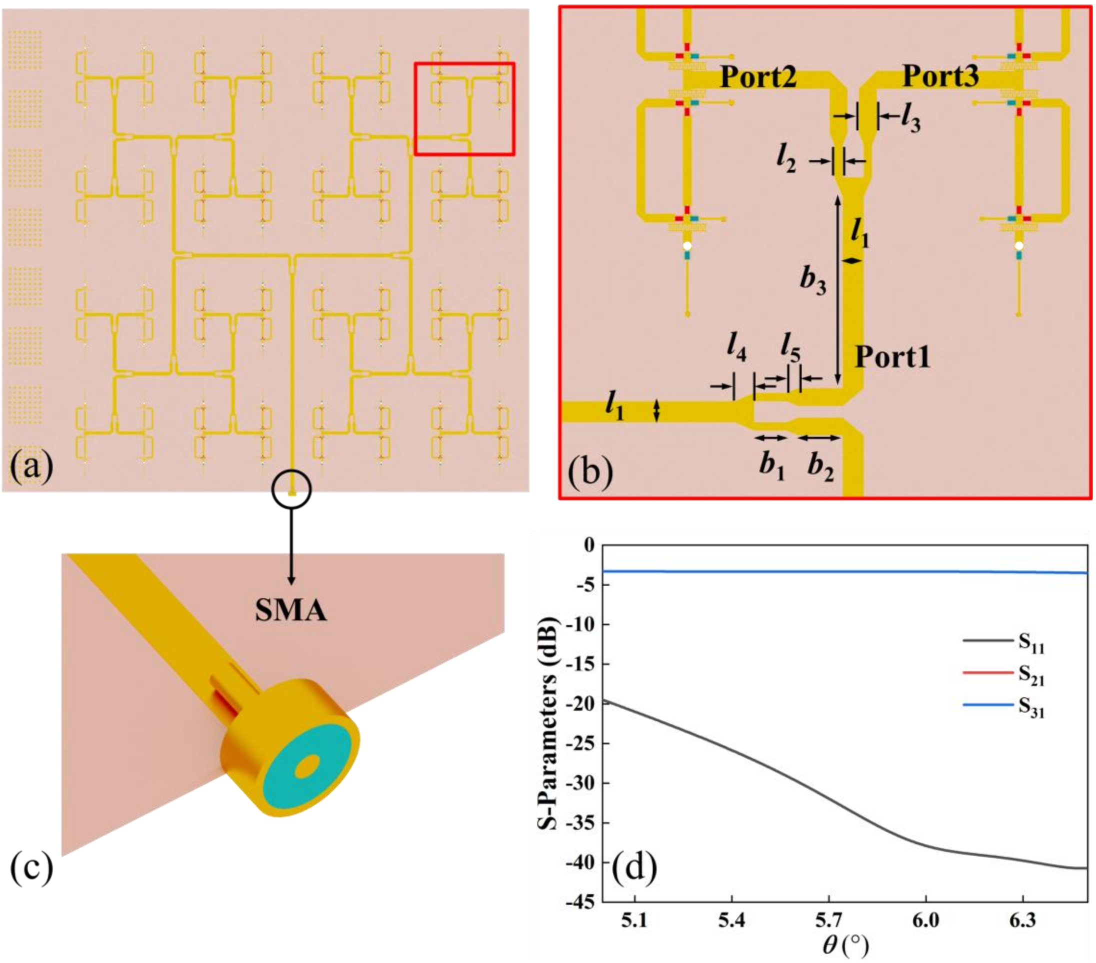

Figure S2. a) The bottom power division network and PSN. b) The detailed structure of the power division network, and l_1 = 1.9mm, l_2 = 0.75mm, l_3 = 1.6mm, l_4 = 1.7mm, l_5 = 1.06mm, b_1 = 2.9375mm, b_2 = 4mm, b_3 = 17.3mm. c) The detailed structure of the SubMiniature version A (SMA). d) The S_{11}, S_{21}, and S_{31} of the power division network.

All PSNs are interconnected via a 1-64 power divider. The excitation of all meta-atoms with equal amplitude and the same phase is achieved through the use of an SMA. The performance of the power divider was verified through simulation. It was observed that S_{11} is less than -20dB within the operating band, while S_{21} and S_{31} exhibited a precise equality of approximately -3.3dB, exhibiting minimal loss.

Note 3. The surface currents of the meta-atom

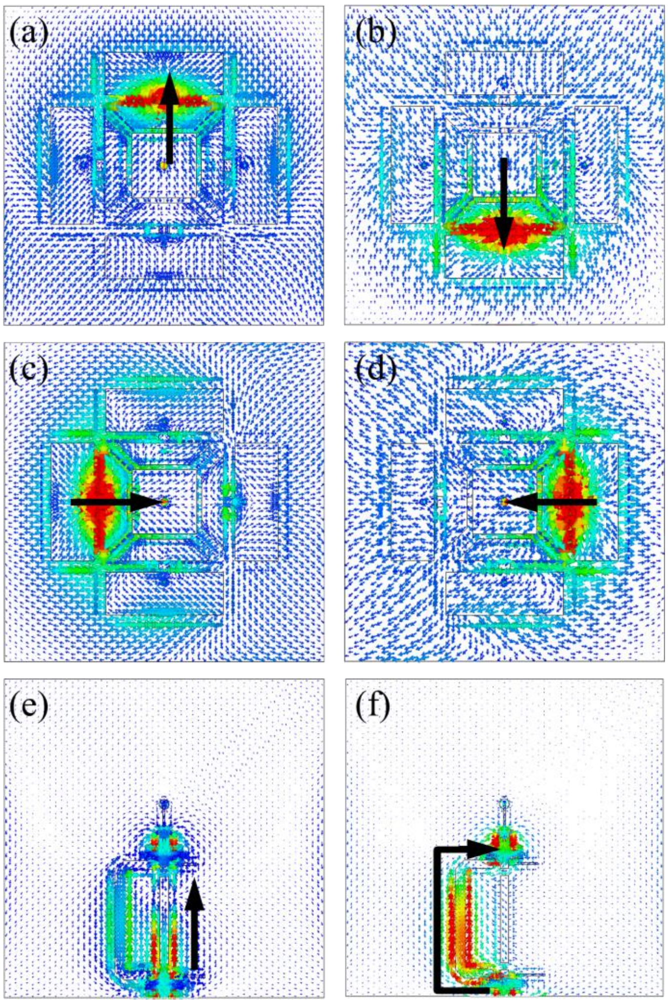

Figure S3. The simulated surface currents at 5.7GHz when a) $Vy_1 = +v_1$, b) $Vy_2 = +v_1$, c) $Vx_1 = +v_1$, d) $Vx_2 = +v_1$, e) $V_1 = -v_0$, and f) $V_1 = +v_0$.

By varying the varactor in different directions, a strong surface current is formed in different directions, as shown in Figure S3. The antenna radiates *x*-polarized EM waves when the varactor in the *x*-direction is conducting, and *y*-polarized EM waves when the varactor in the *y*-direction is conducting. Furthermore, the surface current is reversed by modifying the diode conduction in the +*y* and -*y* directions. Hence, an intrinsic phase shift of 180° can be obtained due to the inversion of the relative spatial position[1].

When $V_1 = -v_1$, the phase delay is 0°, and the phase delay is 90° when $V_1 = +v_1$. As a result, the PSN on the back layer achieves a 90° phase shift. However, when a

specific microstrip line is activated, a strong surface current is also induced on the other microstrip line. Nevertheless, due to the diode obstruction, the surface current can only oscillate and cannot propagate to the feeding hole.

Note 4. The radiation performances of *x*-polarization

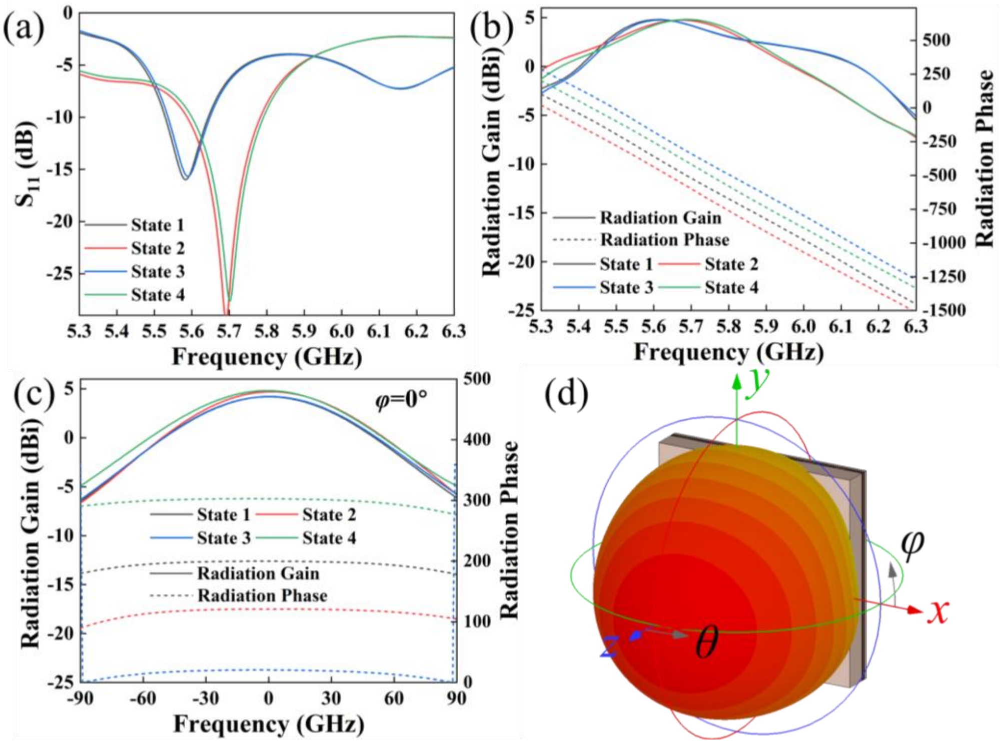

Figure S4. a) The radiation return loss S_{11}. b) The radiation gain and phase varying with frequency at θ=0° and φ=0°. c) The radiation gain and phase varying with θ at φ=0°. d) The 3-D radaiton pattern.

The anisotropic design of the meta-atom ensures that the radiation performance of the *x*-polarization is identical to that of the *y*-polarization. Therefore, the modulation of *x*- and *y*-polarisation is identical, and subsequent space-time coding will also utilize *y*-polarization as an illustrative example.

Note 5. The effect of the number of coding states on +1st harmonic energy

It is assumed that the meta-atom possesses 2^k (k=1, 2,3,…) coding phase states. The phase difference between neighboring phase states is $2\pi/2^k$. For the sake of

simplicity, the +1st harmonic will be used as a basis for discussion, with the understanding that the results for the -1st harmonic are exactly similar. Assume that the time-coding sequence is 0, $(2\pi/2^k)*1$, $(2\pi/2^k)*2$,…, $(2\pi/2^k)*(2^k-1)$. According to Equation (4), the equivalent coefficients of the +1st harmonic can be expressed as follows:

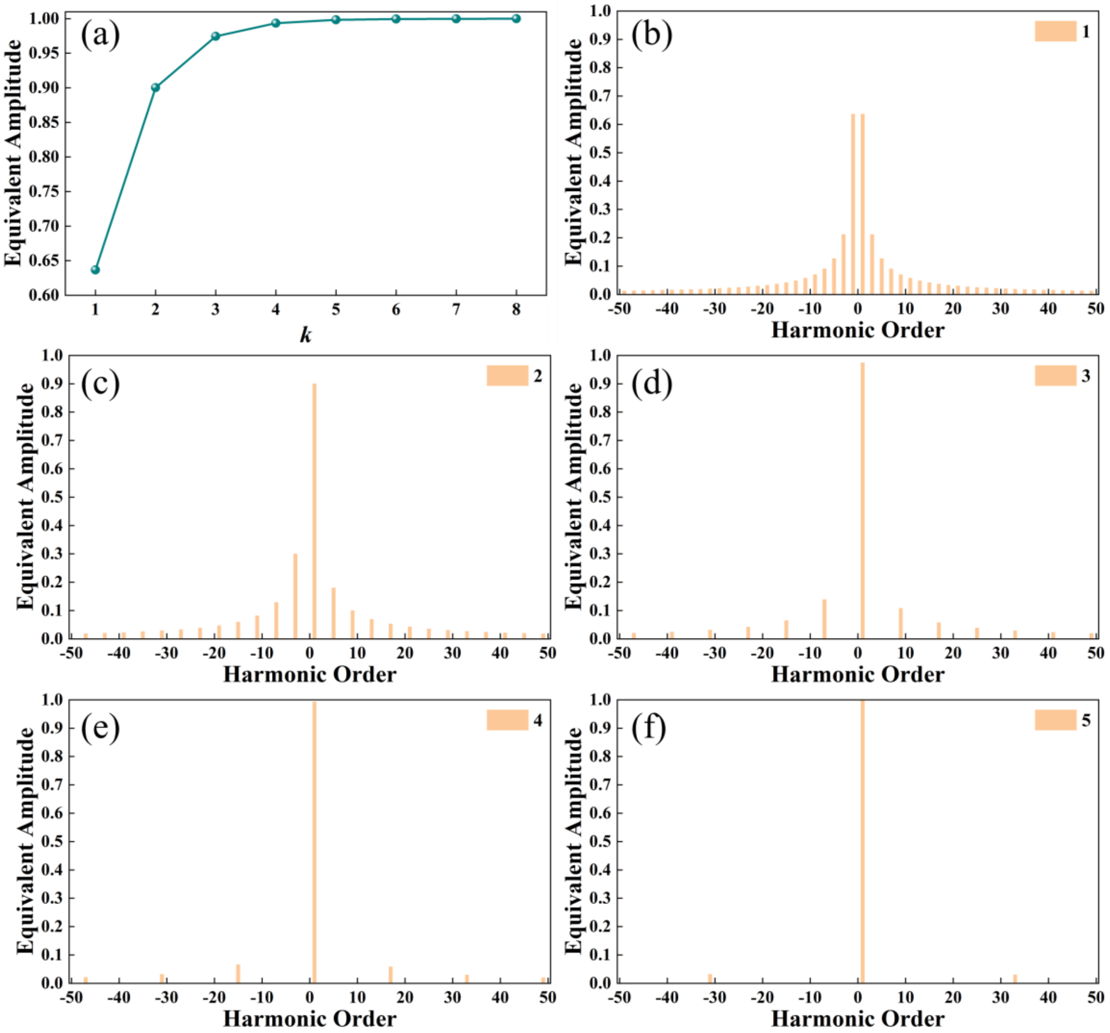

Figure S5. a) The equivalent amplitude of +1st harmonic varies with *k*. The equivalent amplitude of harmonics of each order when b) *k* = 1, c) *k* = 2, d) *k* = 3, e) *k* = 4, and f) *k* = 5.

$$\begin{aligned} r^{+1} &= \frac{\sin(\pi/2^k)}{\pi}\sum_{n=1}^{2^k} exp(j\beta^n - j\pi(2n-1)/2^k) \\ &= \frac{2^k * \sin(\pi/2^k)}{\pi} exp(-j\pi/2^k) \end{aligned} \tag{E1}$$

Therefore, the equivalent amplitude of the +1st harmonic is $2^k * \sin(\pi/2^k)/\pi$. The

equivalent amplitude curve as a function of k is illustrated in Figure S5a, which demonstrates that the function is strictly increasing with respect to k. The equivalent amplitude is greater than 0.9 when $k \geq 2$. Figure S5 gives the equivalent amplitudes of the harmonics of each order when k takes values from 1 to 5. Actually, as the value of k increases, the design difficulty and control complexity of the system also increase significantly. Therefore, a value of k equal to 2 or 3 is sufficient.

$$r^{+1} = \frac{\sin(\pi / 2^k) * 2^k}{\pi} exp(j\pi / 2^k) \tag{E2}$$

It is further considered that the order of the coding sequence could be modified to $(2\pi/2^k)*1$, $(2\pi/2^k)*2$,…, $(2\pi/2^k)*(2^k-1)$, 0. The equivalent coefficients of the +1st harmonic can be expressed as equation E2. A comparison of equations E1 and E2 reveals that there is only a phase difference of $2\pi/2^k$ between the two. Therefore, the phase modulation of the +1st harmonic can be achieved by altering the order of the time-coding sequence without affecting its amplitude[2].

Note 6. The radiation power spectra comparisons between sequential coding and stochastic coding, uniform and non-uniform modulation, and different *L*

Here, we select stochastic coding, non-uniform modulation, L = 1000, θ =10°, and SLL= -13.5 dB as the benchmark for comparison. Firstly, for sequential coding, a substantial number of lower-order harmonics are generated, while higher-order harmonics are effectively suppressed. Consequently, the influence of lower-order harmonics on the center frequency can be significant. In order to suppress low-order harmonics, stochastic coding is employed. Nevertheless, if the identical modulation period is employed for each meta-atom, the generated harmonics will have an identical frequency. Consequently, they will superimpose in space, thereby generating greater energy. Therefore, the non-uniform modulation can further suppress the harmonic energy, as shown in Figure S6b. The maximum SBL is reduced by 8.8 dB

by employing non-uniform modulation.

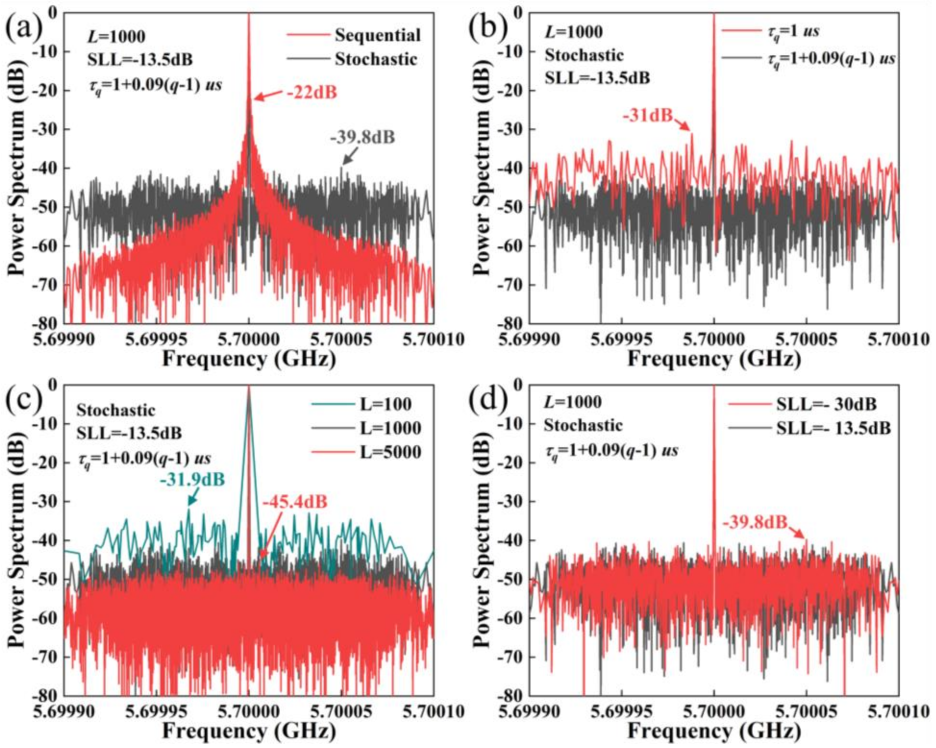

Figure S6. (a) Power spectrum corresponding to stochastic and sequential distributions of time-coding sequences. (b) The power spectrum for modulation periods τ_q = (1+0.09* (q -1)) *us* and τ_q = 1 *us*, respectively. (c) The power spectrum for L = 100, 1000 and 5000. (d) The power spectrum for SLL = -13.5dB and SLL = -30 dB.

Furthermore, the maximum SBL at varying L values was evaluated. As L increases, the maximum SBL decreases, as shown in Figure S6c. This is because an increase in L results in the distribution of energy over a greater number of frequencies. Nevertheless, an excessively large L can impede control and diminish control accuracy. Accordingly, the value of L was set at 1000 for the design. The maximum SBL remains relatively consistent for different SLLs, as shown in Figure S6d. In conclusion, SBL was substantially suppressed by stochastic coding and non-uniform modulation without the use of optimization algorithms.

Note 7. The accuracy of equivalent amplitudes and phases with different *L*

$$r^0 = \sum_{n=1}^{L} \frac{a^n exp(j\beta^n)}{L} \tag{E3}$$

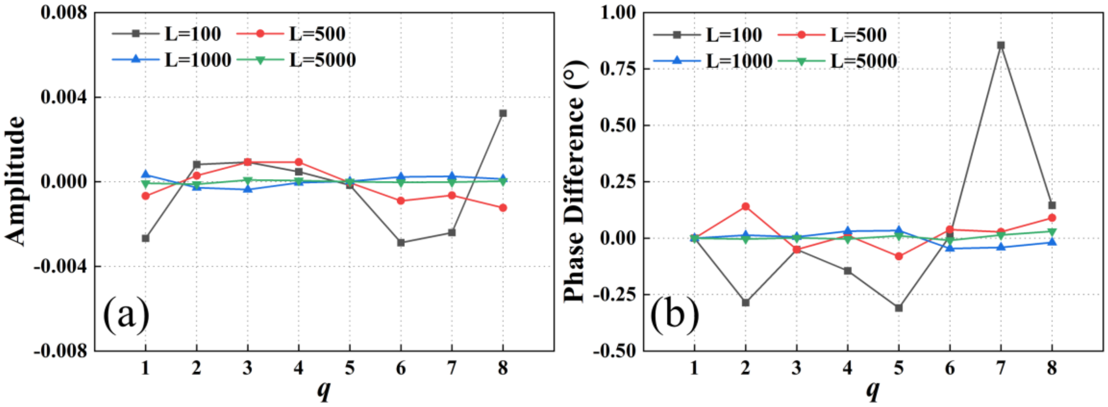

Figure S7. a) The amplitude difference between the actual generated amplitude and the target amplitude for different *L*. b) The phase difference between the actual generated phase and the target phase for different *L*.

The effect of *L* on the accuracy of the amplitude is first discussed. Assuming $\beta^n = 0$, the smallest equivalent amplitude that can be obtained for the time-coding of amplitudes 0 and 1 is 1/*L*. Therefore, an increase in *L* will result in a corresponding improvement in the accuracy of the obtained equivalent amplitude. In this example, we consider SLL equal to -30 dB and a scanning angle of 10°. The discrepancy between the equivalent amplitude and phase and the target amplitude and phase at varying *L* is calculated. The discrepancy is significant when *L*=100, whereas, for *L*=1000 and 5000, the discrepancy is minimal. In order to reduce the complexity of the design and modulation, the value of *L* = 1000 was selected for both the design and the measurement.

Note 8. The analysis of SLL on power efficiency

The reduction of SLL requires a specific gain distribution, which consequently results in an increased number of ‘none’ states within the time-coding sequence. Consequently, the radiation gain decreases as the SLL decreases. Therefore, this is

primarily due to the reduced energy of radiation and is unrelated to harmonics.

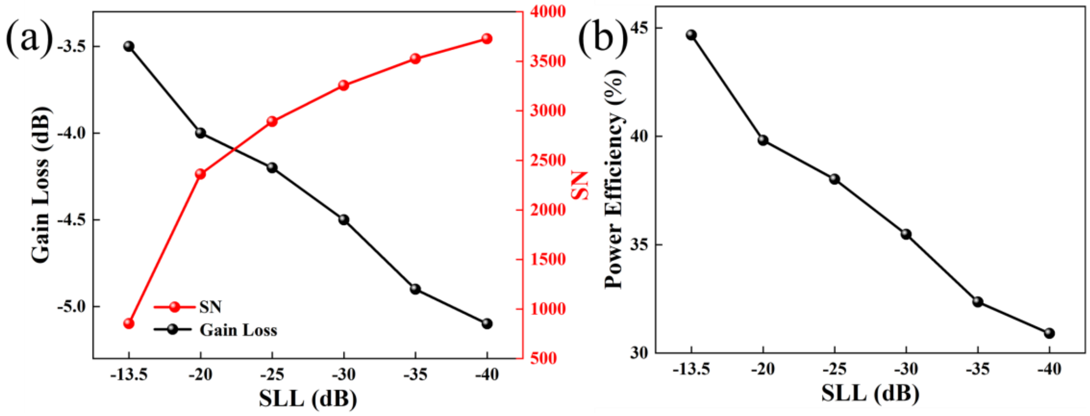

Figure S8. a) The gain loss and SN varying with SLL when θ = 10°. b) The power efficiency varying with SLL when θ = 10°.

It is worth noting that a case of θ = 0° and SLL = -13.5 dB represents an equal amplitude and in-phase excitation with no time modulation. A gain loss of 3.5 dB is observed when θ = 10° and SLL = -13.5 dB. This is not attributable to variations in SLL, but rather to loss resulting from θ. In other words, gain loss essentially arises from time modulation, which encompasses the influences of both scanning angle and SLL. When the scanning angle is not 0°, a specific phase gradient of the array is required. This results in a corresponding maximum current amplitude of less than 0.707, which causes a loss of approximately 3.5 dB, as shown in Figure 4b. Furthermore, lower SLL requires more ‘none’ states, resulting in greater gain loss. In addition, the gain decreases as the scanning angle increases.

$$\eta = 10\log(\delta) \tag{E4}$$

where η and δ denote power efficiency and gain loss, respectively.

In order to evaluate the trade-offs between SLL reduction and gain, θ = 10° is chosen for discussion here without loss of generality. To quantify the relationship between SLL and power efficiency, we give the relationship between gain loss, SLL, and the sum of the number (SN) of ‘none’ states in the time-coding sequence of each meta-atom, as shown in Figure S9a. The power efficiency is defined as in Equation E4. As the SLL decreases, the increasing number of ‘none’ states results in an

increase in gain loss and a concomitant decrease in power efficiency.

Note 9. Improving beam scanning by increasing the number of meta-atoms

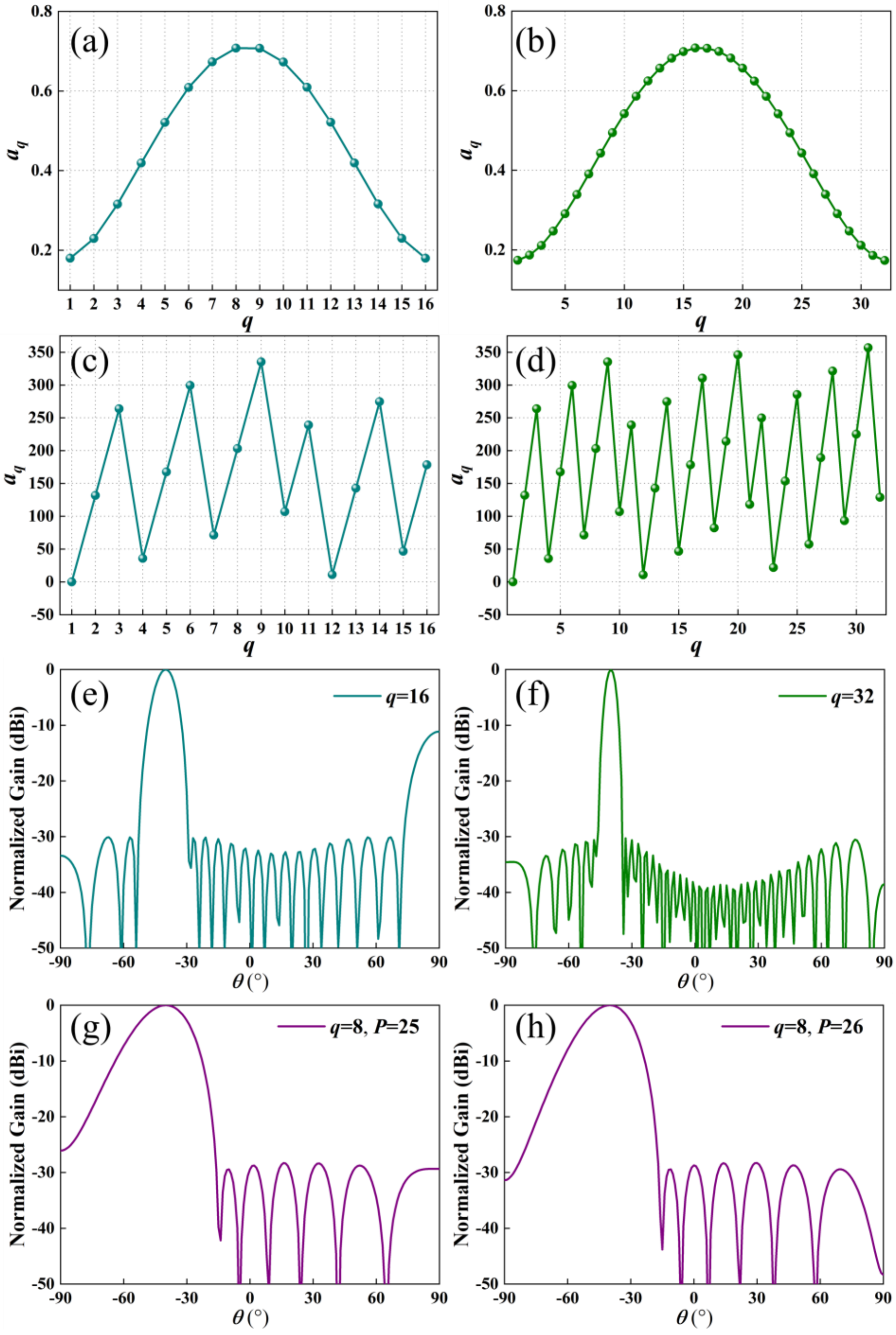

Figure S9. The equivalent amplitude distributions for scanning angle 40° with a) q =16 and b) q=32. The equivalent phase distributions for scanning angle 40° with c) q =16 and d) q=32. The radiation patterns of e) q =16 and f) q=32. The simulated results of beam scanning when SLL = -30 dB, q = 8 and g) P = 25 h) P = 26.

For SLL = -30 dB and q = 8, the performance at a scanning angle of 40° is not good, primarily due to two reasons. The first issue is that the number of meta-atoms is insufficient, which is the primary reason. The second issue is that the period of the meta-atoms is too large. Figure S9 illustrates the simulation results after the number of meta-atoms is increased. A performance improvement is observed when the number of meta-atoms is increased to 16. Furthermore, the requirement of beam scanning with low SLL is entirely fulfilled when the number is increased to 32. In addition, beam scanning performance can also be improved by reducing the period of the meta-atom. When the meta-atom period is reduced from 30 mm to 26 mm, the beam scanning performance can be significantly improved without increasing the number of meta-atoms, as shown in Figure S9h.

Note 10. Beam scanning with lower SLLs

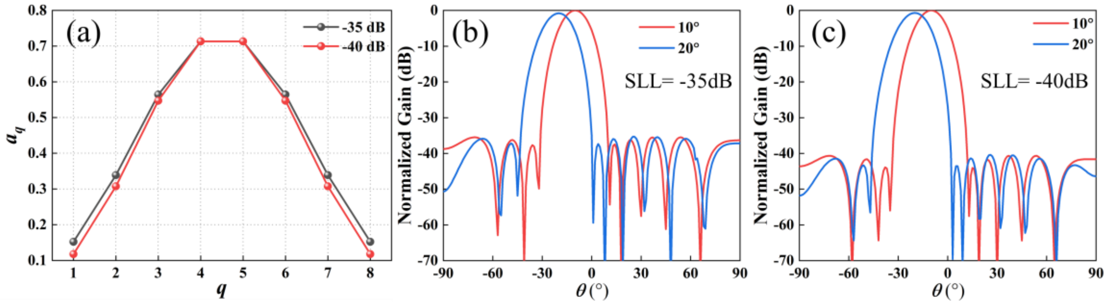

Figure S10. The equivalent amplitude distributions for SLL = -35 dB and -40 dB. The simulated results of beam scanning when b) SLL = -35 dB and c) SLL = -40 dB.

The required amplitude distributions for different SLLs are shown in Figure S10. The phase distribution is the same as in Figure 4c for different scan angles. Further optimization of the amplitude distribution can obtain lower SLL with a value below -40 dB. Nevertheless, the scanning angle is constrained by the limitations of the meta-atom size and the number of array elements. Consequently, as the SLL is reduced, the scanning angle correspondingly becomes smaller. Therefore, scanning angles of 10° and 20° are verified here, as shown in Figure S10b, c.

Note 11. The effect of the number of coding states on reflection reduction

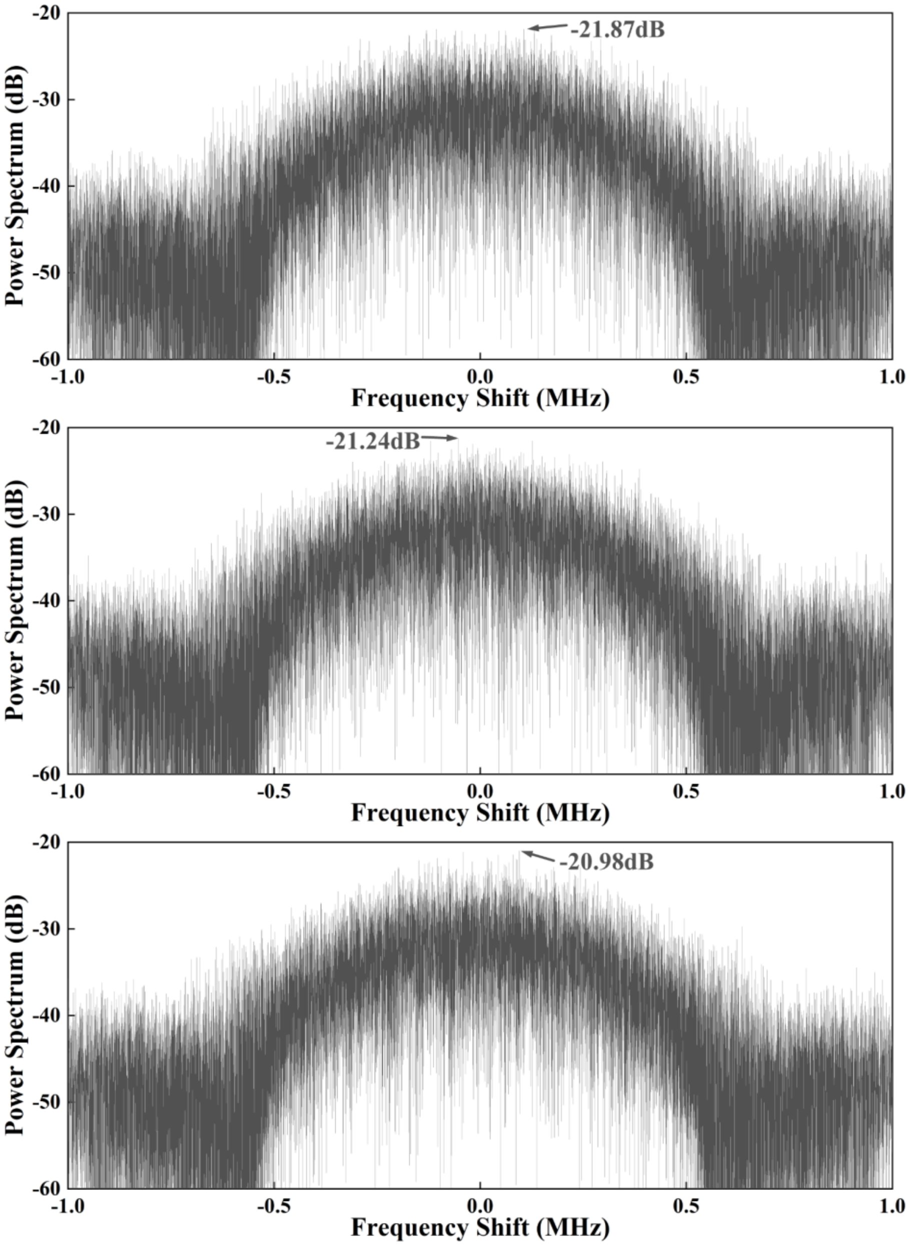

Figure S11. The simulated power spectrum with $L_q = 986 + 2*(q-1)$ at $\theta = 0°$ for a) 1-bit, b) 2-bit, and c) 3-bit.

The maximum reflection energies of 1-bit, 2-bit, and 3-bit are -21.87dB, -21.24dB, and -20.98dB respectively. The number of coding states has a negligible impact on reflection reduction in the context of simulation results. Nevertheless, in practical applications, errors associated with the meta-atom phase can potentially diminish the

efficacy of reflection reduction for a greater number of coding states. In addition, too many coding states increase the complexity of modulation.

References

[1] J. Hu, Z. Hao, *IEEE Trans. Antennas Propag.* **2018**, *66*, 4986.

[2] L. Jiang, Y. Li, H. Yang, L. Zheng, H. Chen, W. Wang, Z. Qin, M. Yan, J. Jiang, Z. Zhu, Y. Meng, J. Wang, S. Qu, *IEEE Trans. Microw. Theory Tech.* **2024**, doi: 10.1109/TMTT.2024.3423007.
